# PI3K‐AKT‐FOXO1 pathway targeted by skeletal muscle microRNA to suppress proteolytic gene expression in response to carbohydrate intake during aerobic exercise

**DOI:** 10.14814/phy2.13931

**Published:** 2018-12-11

**Authors:** Lee M. Margolis, Claire E. Berryman, Nancy E. Murphy, Christopher T. Carrigan, Andrew J. Young, John W. Carbone, Stefan M. Pasiakos

**Affiliations:** ^1^ Military Nutrition Division U.S. Army Research Institute of Environmental Medicine Natick Massachusetts; ^2^ Oak Ridge Institute of Science and Education Oak Ridge Tennessee; ^3^ Department of Nutrition, Food, and Exercise Sciences Florida State University Tallahassee Florida; ^4^ School of Health Sciences Eastern Michigan University Ypsilanti Michigan

**Keywords:** miR‐206, miR‐486, muscle protein breakdown, muscle protein synthesis, myomiR, whey protein

## Abstract

Ingesting protein and carbohydrate together during aerobic exercise suppresses the expression of specific skeletal muscle microRNA and promotes muscle hypertrophy. Determining whether there are independent effects of carbohydrate and protein on microRNA will allow for a clearer understanding of the mechanistic role microRNA serve in regulating skeletal muscle protein synthetic and proteolytic responses to nutrition and exercise. This study determined skeletal muscle microRNA responses to aerobic exercise with or without carbohydrate, and recovery whey protein (WP). Seventeen males were randomized to consume carbohydrate (CHO; 145 g; *n *=* *9) or non‐nutritive control (CON;* n *=* *8) beverages during exercise. Muscle was collected before (BASE) and after 80 min of steady‐state exercise (1.7 ± 0.3 V̇O_2_ L·min^−1^) followed by a 2‐mile time trial (17.9 ± 3.5 min; POST), and 3‐h into recovery after consuming WP (25 g; REC). RT‐qPCR was used to determine microRNA and mRNA expression. Bioinformatics analysis was conducted using the mirPath software. Western blotting was used to assess protein signaling. The expression of six microRNA (miR‐19b‐3p, miR‐99a‐5p, miR‐100‐5p, miR‐222‐3p, miR‐324‐3p, and miR‐486‐5p) were higher (*P *<* *0.05) in CHO compared to CON, all of which target the PI3K‐AKT, ubiquitin proteasome, FOXO, and mTORC1 pathways. p‐AKT^T^ ^hr473^ and p‐FOXO1^Thr24^ were higher (*P *<* *0.05) in POST CHO compared to CON. The expression of *PTEN* was lower (*P *<* *0.05) in REC CHO than CON, while *MURF1* was lower (*P *<* *0.05) POST CHO than CON. These findings suggest the mechanism by which microRNA facilitate skeletal muscle adaptations in response to exercise with carbohydrate and protein feeding is by inhibiting markers of proteolysis.

## Introduction

Protein and carbohydrate ingestion modulate cellular mechanisms regulating skeletal muscle mass in response to exercise (Churchward‐Venne et al. 2012; Pasiakos 2012; Stokes et al. 2018). Consuming high‐quality protein, such as whey, increases activation of the mechanistic target of rapamycin complex 1 (mTORC1) pathway, eliciting a greater muscle protein synthetic response compared to exercise alone (Kimball and Jefferson 2004, 2006). Carbohydrate ingestion may contribute to muscle accretion through antiproteolytic effects, reducing muscle protein breakdown by inhibiting the forkhead box O 1 (FOXO1) pathway through an insulin‐dependent mechanism (Roy et al. 1997; Biolo et al. 1999; Greenhaff et al. 2008; Abdulla et al. 2016). Several investigations have reported that the expression of specific microRNA (miRNA) linked to governing mTORC1 are also acutely altered in response to exercise (Drummond et al. 2008; Rivas et al. 2014; Zacharewicz et al. 2014; Fyfe et al. 2016; D'Souza et al. 2017b) and protein feeding (Drummond et al. 2009; Camera et al. 2016; Margolis et al. 2017). However, few studies have been able to capture downstream modifications in biological processes targeted by altered skeletal muscle miRNA in humans. Our laboratory (Margolis et al. 2017) recently reported that specific miRNA (miR‐1, miR‐206, miR‐208, and miR‐499) are diminished and inversely associated with muscle protein synthesis after aerobic exercise when essential amino acids and carbohydrate are consumed together during exercise.

miRNA control biological processes through inhibition, binding to target mRNA to repress protein translation (Kong et al. 2008). The posttranscriptional control that miRNA exert on target mRNA make them prospective regulators of exercise and diet‐induced alterations in skeletal muscle. Rodent and cell culture models have shown that the overexpression of miR‐1, miR‐99a, miR‐99b, miR‐100‐5p, and miR‐133 blunt rates of protein synthesis by directly or indirectly inhibiting mTORC1, resulting in lower muscle fiber size and mass (McCarthy and Esser 2007; Elia et al. 2009; Hua et al. 2012; Jin et al. 2013; Wei et al. 2013). Conversely, the overexpression of miR‐221, miR‐222, and miR‐486 inhibit phosphatase and tensin homolog (PTEN), allowing for increased AKT and diminished FOXO1, inhibiting proteolysis and resulting in muscle growth (Chun‐Zhi et al. 2010; Small et al. 2010). However, the function of these miRNA in humans is not well defined. Particularly, much remains unknown regarding independent effects of protein and carbohydrate intake on modulation of miRNA responses to exercise in humans. Understanding how protein and carbohydrate alter miRNA and their downstream targets would offer a new level insight into the mechanism by which manipulation of nutrient intake promotes muscle growth.

The objective of this study was to examine the effects of aerobic exercise with or without carbohydrate ingestion during exercise, and whey protein (WP) feeding during postexercise recovery, on skeletal muscle miRNA. Additionally, this study sought to identify the pathways that differentially expressed miRNA targets in response to carbohydrate supplementation. We hypothesized, independent of carbohydrate intake, that miRNA associated with the regulation of mTORC1 would be downregulated, and that miRNA differentially expressed in response to carbohydrate intake would target proteolysis via the FOXO1 pathway.

## Materials and Methods

### Participants

Seventeen free‐living, recreationally active (programmed physical activity 2–4 days/week) males completed this randomized, parallel, single‐blinded, placebo‐controlled study. Participants were in good health and refrained from consuming alcohol, caffeine, or dietary supplements, and nicotine products during the study. Data presented in this manuscript were collected as part of a larger study examining the impact of high‐altitude exposure and underfeeding on skeletal muscle mass, intramuscular regulators of muscle anabolism and proteolysis, and substrate metabolism (Berryman et al. 2018; Margolis et al. 2018; Young et al. 2018). This manuscript reports observations from only the sea‐level experiments, in which acute miRNA expression and their targets were measured in response to aerobic exercise, with or without carbohydrate, and recovery protein feeding. This study was approved by the Institutional Review Board at the US Army Research Institute of Environmental Medicine (USARIEM, Natick, MA). This study was conducted from May 2016 through August 2016. All testing was conducted at Natick, MA. All participants provided written informed consent before participation. Forty‐one individuals signed the informed consent. The trial was registered at www.clinicaltrials.gov as NCT02731066.

After obtaining informed consent, participants were randomly assigned to consume either a carbohydrate (CHO; *n *=* *9) or flavor‐matched non‐nutritive control (CON; *n *=* *8) drink during 80 min of steady‐state treadmill exercise (Young et al. 2018). Randomization was computer generated, with 1:1 allocation for the parallel groups. Height was measured to the nearest 0.1 cm with a stadiometer (Seca; Creative Health Products, Plymouth, MI), and body mass was determined to the nearest 0.1 kg using a calibrated digital scale (Model PS6600; Befour Inc., Saukville, WI). Body composition was measured by dual energy X‐ray absorptiometry (DEXA; DPX‐IQ; GE Lunar Corp., Madison, WI). For 7 days before testing, participants followed dietary instructions for a weight maintenance diet, consuming 1.0 g·kg^−1^·d^−1^ protein, with 45–65% total energy from carbohydrate, and 20–35% total energy from fat, which was verified by study research dietitians using 24 h recalls. There were no differences in participant characteristics or energy and macronutrient intake between groups (Table 1).

**Table 1:** Volunteer characteristics^a^

Characteristic	CHO (*n *=* *9)	CON (*n *=* *8)
Age (years)	22 ± 2	25 ± 8
Height (cm)	178 ± 9	175 ± 6
Weight (kg)	84 ± 13	80 ± 16
Body mass index (kg·m^−2^)	27 ± 3	26 ± 4
Fat‐free mass (kg)	63 ± 8	59 ± 10
Fat mass (kg)	19 ± 6	18 ± 8
Body fat (%)	23 ± 5	23 ± 7
VO_2peak_ (L·min^−1^)	4.3 ± 0.7	4.0 ± 0.6
bEnergy (kcal·d^−1^)	2491 ± 525	2414 ± 591
bProtein (g·kg^−1^·d^−1^)	1.2 ± 0.4	1.3 ± 0.4
bCarbohydrate (g·kg^−1^·d^−1^)	3.9 ± 0.9	4.0 ± 1.1
bFat (g·kg^−1^·d^−1^)	1.1 ± 0.3	1.1 ± 0.4

CHO, carbohydrate; CON, control.

a Values are means ± SD.

b Energy and macronutrient intake determined using 24 h food recalls.

### Experimental design

**Figure 1:**
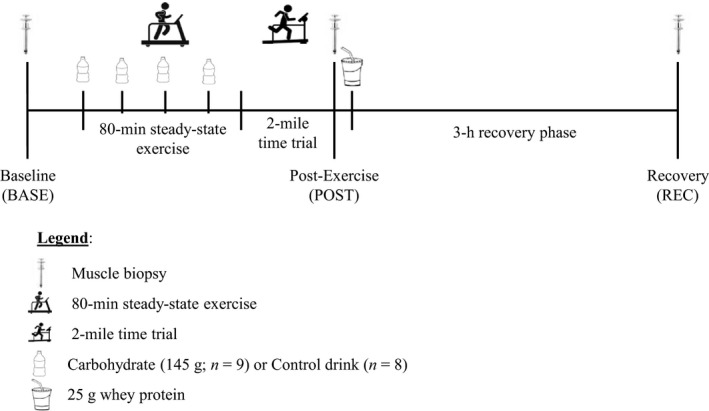
Study design.

Following a 10‐h overnight fast, a biopsy was collected from the vastus lateralis to capture rested/postabsorptive muscle basal state (BASE; Fig. 1). Participants then completed 80 min of steady‐state treadmill exercise, walking at a fixed speed and grade that elicited 40% of VO_2_peak. After completing 80 min of walking, participants were allowed 5‐min standing rest, and then they completed a 2‐mile time trial in which they attempted to run or walk at the fastest pace that they could maintain. Participants consumed either a carbohydrate (CHO; *n *=* *9) or flavor‐matched non‐nutritive control (CON; *n *=* *8) drink at 0‐, 20‐, 40‐, and 60‐min during the steady‐state portion of the exercise bout (Young et al. 2018). The carbohydrate drink contained 145 g of fructose and glucose (62.25 g fructose + 79.75 g glucose; 0.8 fructose‐to‐glucose ratio) to achieve an average ingestion rate of 1.8 g min^−1^. Indirect calorimetry (True Max 2400, Parvo Medics, Sandy, UT) was performed during the steady‐state exercise to ensure V̇O_2_ was within ±5% of the target V̇O_2_. Steady‐state exercise was metabolically matched between the groups (CHO: 1.78 ± 0.22 V̇O_2_ L·min^−1^; 713 ± 86 kcal, CON: 1.63 ± 0.26 V̇O_2_ L·min^−1^; 651 ± 104 kcal; *P *>* *0.05). There was no difference between the groups in the time required to complete the 2‐mile time trial (CHO: 17.8 ± 3.9 min, CON: 18.0 ± 3.3 min; *P *>* *0.05). Immediately after the time trial, a postexercise (POST) muscle biopsy was obtained. Participants then consumed a 25 g bolus of whey protein drink (Isopure^®^ Zero Carb; Isopure Co., Hauppauge, NY). After a 3‐h recovery period, a final muscle biopsy was obtained (REC).

### microRNA and mRNA expression

Total RNA was isolated from 20 mg muscle samples using TRIzol reagent (ThermoFisher, Waltham, MA). RNA quantity and quality were assessed using a Nanodrop ND‐2000 spectrophotometer (Nanodrop, Wilmington, DE). For miRNA analysis, equal amounts of total RNA (250 ng) were reverse‐transcribed using the TaqMan^®^ microRNA RT kit (Applied Biosystems, Foster City, CA) with the miRNA‐specific stem‐loop reverse transcript (RT) primers pooled in 1X Tris‐EDTA (TE) buffer for a final dilution of 0.05× for each miRNA RT primer. For mRNA, equal amounts (500 ng) of total RNA were reverse‐transcribed using a iScript™ Advanced cDNA Synthesis Kit (Bio‐Rad). Both mRNA and miRNA reverse transcription was conducted using a T100™ Thermal Cycler (Bio‐Rad). Amplifications were performed using a StepOnePlus Real‐Time PCR System (Applied Biosystems). Samples were run in 15 *μ*L reactions in duplicate using TaqMan^®^ Universal PCR MasterMix (2X), no UNG (Applied Biosystems) and commercially available miRNA probes (miR‐1‐3p, miR‐26a‐5p, miR‐29a‐3p, miR‐99a‐5p, miR‐99b‐5p, miR‐100‐5p, miR‐103, miR‐133a‐3p, miR‐133b, miR‐206, miR‐208b, miR‐221‐5p, miR‐222‐3p, miR‐324‐3p, miR‐378‐5p, miR‐451a, miR‐486‐5p, and miR‐499‐3p; Applied Biosystems), and 10 *μ*L reactions using iTaq™ Universal SYBR^®^ and commercially available primers for mRNA (*PTEN, AKT1*,* FOXO1, MURF1*; Bio‐Rad). miRNA included in the present analysis were chosen based on their potential to regulate pathways governing skeletal muscle protein synthesis and proteolysis. All miRNA were normalized to RNU44, and all mRNA were normalized to the geometric mean of *B2M* and *β‐ACTIN*. Fold changes for miRNA and mRNA were calculated using the ΔΔ cycle threshold (ΔΔCT) method (Pfaffl 2001) and expressed as fold change relative to BASE for each group. *MURF1* data from the CON group was previously published (Margolis et al. 2018) to assess changes in response to exercise and protein feeding at sea level, acute high altitude (3 h), and chronic high altitude (21 days). No comparison of the CHO and CON groups has been published for the measures included in the current manuscript.

### Bioinformatics analysis

miRNA that were differentially expressed between CHO and CON were uploaded to DNA Intelligent Analysis (DIANA)‐miRPath 3.0 (Alexander Fleming Biological Sciences Research Center [BSRC], Athens, Greece; http://diana.cslab.ece.ntua.gr) to determine common molecular pathways that these miRNA regulate. Kyoto Encyclopedia of Genes and Genomes (KEGG; http://www.genome.jp/kegg/) pathways were identified using only experimentally verified targets from TarBase 7.0 (Alexander Fleming BSRC). Pathways specific to disease were excluded from results.

### Intracellular signaling

Phosphorylation status were determined from western blots as previously described (Margolis et al. 2018). Muscle samples (~20 mg) were homogenized in ice‐cold homogenization buffer. Homogenates were centrifuged for 15 min at 10,000***g*** at 4°C. Supernatant (lysate) was collected and protein concentrations were determined using 660 nm Protein Assay (ThermoFisher). Muscle lysates were solubilized in Laemmli buffer, with equal amounts of total protein (15 *μ*g) and separated by SDS‐PAGE using precast Tris‐HCl gels (Bio‐Rad). Proteins were transferred to polyvinylidene fluoride membranes and exposed to commercially available primary antibodies specific to p‐mTOR^Ser2448^, p‐rpS6^240/244^, p‐Akt^Ser473^, p‐FOXO1^Thr24^ (Cell Signaling Technology, Danvers, MA) overnight at 4°C. Secondary antibody (anti‐rabbit IgG conjugate with horseradish peroxidase; Cell Signaling Technology) and chemiluminescent reagent (Pierce Biotechnology, Rockford, IL) were applied to label primary antibodies. Blots were quantified using a phosphoimager (ChemiDoc XRS; Bio‐Rad) and Image Lab software (Bio‐Rad). Heat shock protein 90 (HSP90) was used to confirm equal amounts of protein were loaded per well. All phosphorylation data were presented as fold change relative to BASE phosphorylation for each group. Data from the CON group were previously published (Margolis et al. 2018) to assess changes in response to exercise and protein feeding at sea level, acute high altitude (3 h), and chronic high altitude (21 days). No comparison of the CHO and CON groups has been published for the measures included in the current manuscript.

### Statistical analysis

Mixed model repeated measured ANOVA was used to determine impact of time (BASE, POST, REC) and supplement (CHO and CON) on miRNA and mRNA expression and phosphorylation status of intramuscular signaling markers. Bonferroni adjustments for multiple comparisons were performed if significant interactions were observed. All data are presented as mean ± SD. The *α* level for significances was set at *P *<* *0.05. Data were analyzed using IBM SPSS Statistics for Windows Version 22.0 (IBM Corp. Armonk, NY).

## Results

**Figure 2:**
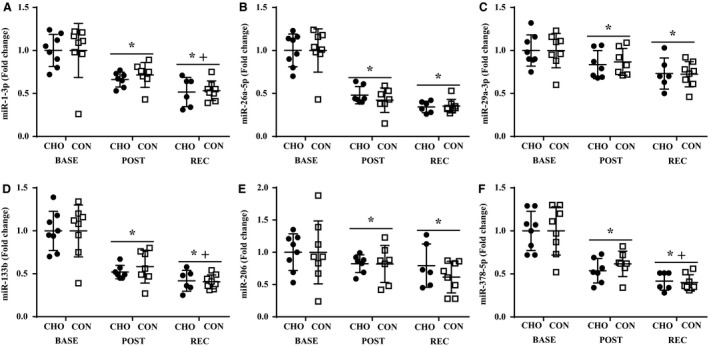
Expression miR‐1‐3p (A), miR‐26a‐5p (B), miR‐29a‐5p (C), miR‐133b (D), miR‐206 (E), miR‐206 (D), and miR‐378‐5p (F). *Different from BASE,* P *<* *0.05. ^+^Different from POST,* P *<* *0.05. BASE, baseline; POST, postexercise; REC, recovery; CHO, carbohydrate; CON, control.

**Figure 3:**
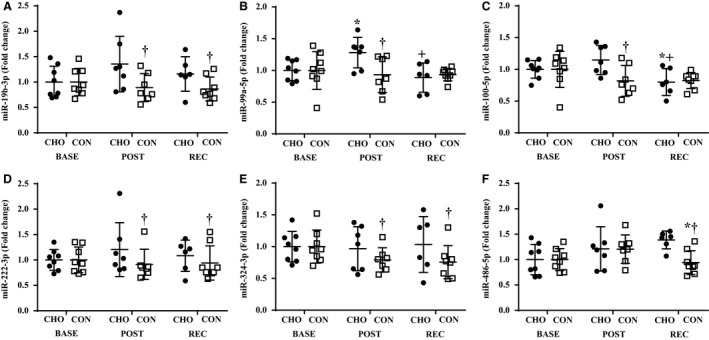
Expression miR‐19b‐3p (A), miR‐99a‐5p (B), miR‐100‐5p (C), miR‐222‐3p (D), miR‐324‐3p (E), and miR‐486‐5p (F) at BASE, POST, and REC. *Different from BASE,* P *<* *0.05. ^+^Different from POST,* P *<* *0.05. ^†^Different from CHO,* P *<* *0.05. BASE, baseline; POST, post exercise; REC, recovery; CHO, carbohydrate; CON, control.

miR‐1‐3p, miR‐26a‐5p, miR‐29a‐3p, miR‐133b, miR‐206, and miR‐378‐5p, were lower (*P *<* *0.05) at POST and REC compared to BASE, regardless of group (Fig. 2A–F). Additionally, miR‐1‐3p, miR‐133b, and miR‐378‐5p were lower (*P *<* *0.05) at REC compared to POST, independent of group (Fig. 2A,D and F). Overall, miR‐19b‐3p, miR‐222‐3p, and miR‐324‐3p were lower (*P *<* *0.05) in CON compared to CHO, with no effect of time (Fig. 3A,D and E). Time‐by‐group interactions were observed for miR‐99a‐5p, miR‐100‐5p, and miR‐486‐5p (Fig. 3B,C and F). Specifically, miR‐99a‐5p POST was higher (*P *<* *0.05) than BASE and REC in CHO (Fig. 3B). miR‐100‐5p was lower (*P *<* *0.05) at REC compared BASE and POST in CHO (Fig. 3C). Both miR‐99a‐5p and miR‐100‐5p were lower (*P *<* *0.05) at POST in CON versus CHO (Fig. 3B and C). miR‐486‐5p REC was lower (*P *<* *0.05) than BASE in CON, and CON was lower (*P *<* *0.05) than CHO at REC (Fig. 3F). No impact of time or group was observed for miR‐99b‐5p, miR‐103, miR‐133a‐3p, miR‐451a, and miR‐499‐3p. miR‐221‐5p was measured but did not cross the cycle threshold during the 40 cycle run, so no data were generated for this miRNA.

**Figure 4:**
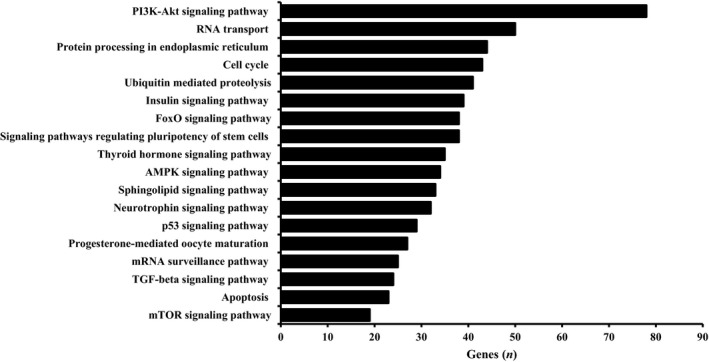
Kyoto Encyclopedia of Genes and Genomes (KEGG) pathways experimentally validated to be targeted by all six miRNA (miR‐19b‐3p, miR‐99a‐5p, miR‐100‐5p, miR‐222‐3p, miR‐324‐3p, and miR‐486‐5p) differentially expressed between carbohydrate (CHO) and control (CON).

Bioinformatics analysis indicated that miR‐19b‐3p, miR‐99a‐5p, miR‐100‐5p, miR‐222‐3p, miR‐324‐3p, and miR‐486‐5p all interact with the PI3K‐AKT signaling pathway (Fig. 4). Downstream targets of PI3K‐AKT, ubiquitin‐mediated proteolysis, FOXO signaling, and mTOR signaling were also identified as pathways targeted by all six miRNA that were differentially expressed between CHO and CON.

**Figure 5:**
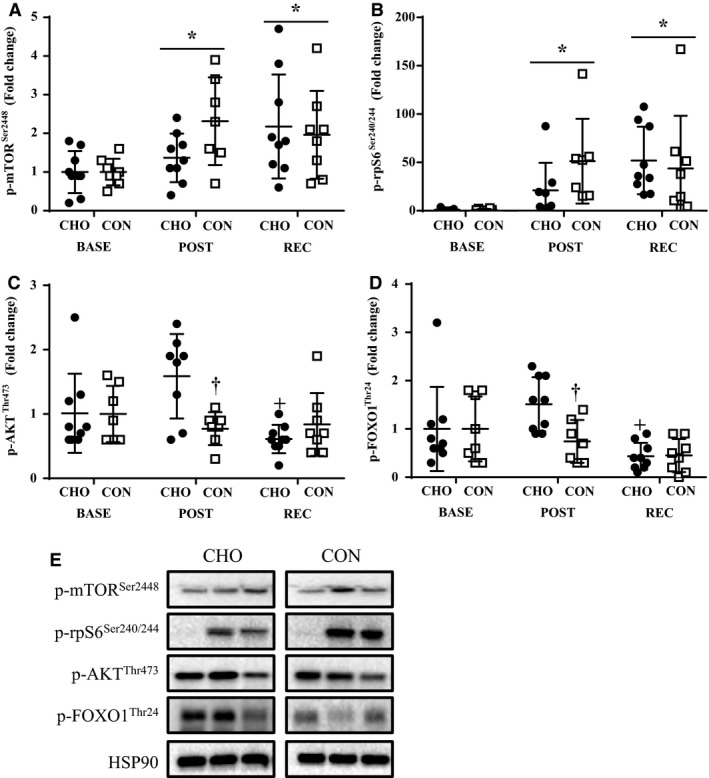
Phosphorylation status p‐mTOR^S^ ^er2448^ (A), p‐rpS6^Ser240/244^ (B), p‐AKT^T^ ^hr473^ (C), and p‐FOXO1^Thr24^ (D). Representative bands (E). *Different from BASE,* P *<* *0.05. ^+^Different from POST,* P *<* *0.05. ^†^Different from CHO,* P *<* *0.05. BASE, baseline; POST, postexercise; REC, recovery; CHO, carbohydrate; CON, control.

**Figure 6:**
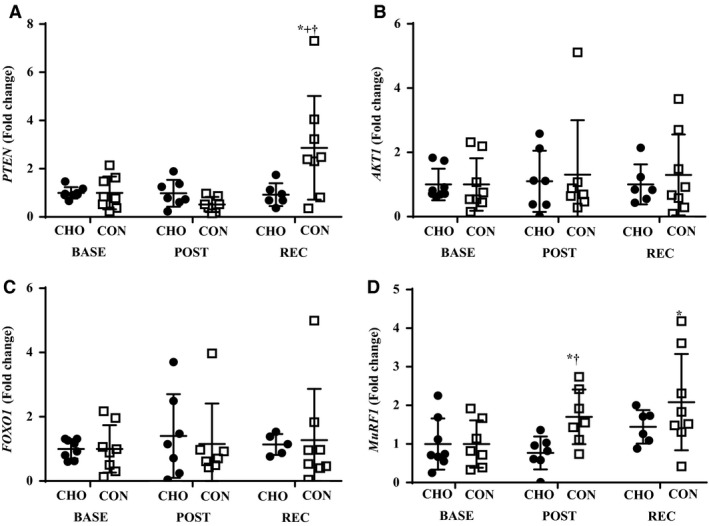
Expression of *PTEN* (A), *AKT1* (B), *FOXO1* (C), and *MURF1* (D). *Different from BASE,* P *<* *0.05. ^+^Different from POST,* P *<* *0.05. ^†^Different from CHO,* P *<* *0.05. BASE, baseline; POST, post exercise; REC, recovery; CHO, carbohydrate; CON, control.

Independent of group, p‐mTOR^Ser2448^ and p‐rpS6^Ser240/244^ were higher (*P *<* *0.05) at POST and REC than BASE (Fig. 5A and B). Time‐by‐group interactions were present in both p‐AKT^Thr473^ and p‐FOXO1^Thr24^, where POST was higher (*P *<* *0.05) than BASE in CHO, and CON was lower (*P *<* *0.05) than CHO at POST (Fig. 5C and D). Time‐by‐group interactions were observed for *PTEN*, with REC being higher (*P *<* *0.05) than BASE and POST in CON, and CON was greater (*P *<* *0.05) than CHO at REC (Fig. 6A). There was no effect of time or group for *AKT1* and *FOXO1* (Fig. 6B and C). Time‐by‐group interactions were observed for *MURF1*, with POST and REC being higher than BASE in CON, and CON was greater than CHO at POST (Fig. 6D).

## Discussion

The main outcome of this study was that miR‐19b‐3p, miR‐99a‐5p, miR‐100‐5p, miR‐222‐3p, miR‐324‐3p, and miR‐486‐5p were more highly expressed in the skeletal muscle following aerobic exercise in CHO compared to CON. Bioinformatics analysis determined that all six of these miRNA target the PI3K‐AKT pathway, ubiquitin proteasome, FOXO, and mTOR signaling pathways. Examination of these pathways identified group differences in markers of proteolysis, but not anabolism. These findings suggest carbohydrate ingestion during aerobic exercise alters miRNA that inhibit skeletal muscle proteolysis with no impact on anabolism.

Of the six miRNA expressed differently in CHO and CON, miR‐19b‐3p, miR‐222‐3p, and miR‐486‐5p have been experimentally validated to inhibit *PTEN* (Schiaffino and Mammucari 2011). In vitro studies demonstrate that overexpression of miR‐19b‐3p, miR‐222‐3p, and miR‐486‐5p diminishes PTEN protein content and increases the phosphorylation status and total protein content of AKT (Chun‐Zhi et al. 2010; Small et al. 2010; Alexander et al. 2011; Xu et al. 2016). Additionally, overexpression of miR‐100‐5p and miR‐324‐3p in cell culture experiments have been found to result in elevated AKT protein content (Jin et al. 2013; Xiao et al. 2017). Increased activation of AKT inhibits FOXO1, a transcription factor mediating expression of the ubiquitin ligases *MURF1* and *ATROGIN‐1* (Bodine et al. 2001; Sandri et al. 2004; Leger et al. 2006). As such, increased AKT total protein content through altered miRNA expression, particularly miR‐486‐5p, in cell culture and rodent studies (Small et al. 2010; Alexander et al. 2011; Xu et al. 2012) results in reduced transcription of these atrophy genes, diminished muscle proteolysis, and increased muscle fiber size (Xu et al. 2012). However, there has been little translational work to determine if there are similar alterations with miRNA and their target pathways in humans. Analysis of the proteolytic pathway in the current study is largely in agreement with past findings in animal and cell culture models (Chun‐Zhi et al. 2010; Small et al. 2010; Xu et al. 2012, 2016). When miR‐19b‐3p, miR‐222‐3p, miR‐324‐3p, and miR‐486‐5p expression is higher in the muscle, there is lower *PTEN* expression, increased activity of p‐AKT^Thr473^, and lower activity of p‐FOXO1^Thr24^ and *MURF1* expression. Higher expression of miRNA that target markers of proteolysis, such as miR‐486‐5p, in the recovery phase of exercise reported here and in previous studies (Camera et al. 2016; Fyfe et al. 2016; D'Souza et al. 2017b), may be favorable to facilitate remodeling, repair, and synthesis of new muscle proteins with exercise.

Independent of carbohydrate, following aerobic exercise with recovery whey protein feeding miR‐1‐3p, miR‐133b, and miR‐206 were lower compared to resting measures. Reductions in these miRNA occurred concurrent with increased mTORC1 signaling. These results are in agreement with previous findings from our laboratory (Margolis et al. 2017) and others (McCarthy and Esser 2007; Drummond et al. 2008; Elia et al. 2009; Rivas et al. 2014; Zacharewicz et al. 2014), indicating that when muscle anabolism is upregulated in response to exercise and protein feeding, miRNA that inhibit the mTORC1 pathway are diminished. Despite findings from the current study corroborating past results from our group and others, conflicting responses in miRNA expression following exercise have been reported (Drummond et al. 2009; Nielsen et al. 2010; Russell et al. 2013; Camera et al. 2016; Fyfe et al. 2016). Discordant results can primarily be attributed to nutrient intake. Specifically, aerobic exercise without nutritional intervention results in increased expression of miR‐1, miR‐133a, miR‐133b, and miR‐206 1 to 3‐hour postexercise (Nielsen et al. 2010; Russell et al. 2013). These differences in miRNA expressions indicate that inclusion of protein feeding immediately after aerobic exercise elicits a unique miRNA expression profile that may potentiate the muscle protein synthetic response compared to aerobic exercise alone.

Overall, results from the current study are consistent with previous investigations showing muscle protein synthetic response is unaffected by carbohydrate ingestion during exercise (Borsheim et al. 2004b; Koopman et al. 2008; Kato et al. 2016), while proteolysis is lower compared to a non‐nutritive control (Roy et al. 1997; Borsheim et al. 2004a; Greenhaff et al. 2008; Glynn et al. 2010). However, to the best of our knowledge, this is the first report in which differences in acute protein synthetic and proteolytic responses to carbohydrate feeding during exercise extend to the miRNA that regulate these pathways. These findings provide new insight into the impact of protein and carbohydrate supplementation on acute alterations in the expression of skeletal muscle miRNA following exercise and their potential regulation of pathways governing muscle accretion. While several investigations have focused on the impact of exercise on skeletal muscle miRNA (Nielsen et al. 2010; Russell et al. 2013; Rivas et al. 2014; Zacharewicz et al. 2014; Fyfe et al. 2016; D'Souza et al. 2017a, 2017b), only a few have explored the role of protein and/or carbohydrate supplementation on modulation of miRNA (Drummond et al. 2009; Camera et al. 2016; Margolis et al. 2017; D'Souza et al. 2018). While the current study shows divergent responses in specific miRNA with or without carbohydrate supplementation, the mechanism that caused these differences is unclear. It is likely that higher expressions in CHO compared to CON were driven by alterations in insulin or glucose concentrations. However, the present study is limited in making this mechanistic distinction. Given the limited information available on nutrient manipulation and miRNA expression, future investigation is warranted to expand our understanding of the mechanistic impact that miRNA may have on the regulation of human skeletal muscle mass.

In conclusion, results from this study with human research participants expands upon previous findings from cell culture and animal research. Specifically, the findings from this study demonstrate that miR‐19b‐3p, miR‐99a‐5p, miR‐100‐5p, miR‐222‐3p, miR‐324‐3p, and miR‐486‐5p were more highly expressed in human skeletal muscle after aerobic exercise during which carbohydrate was consumed compared to after exercise during which no carbohydrate was consumed. These six miRNA target the PI3K‐AKT‐FOXO pathway. Alterations in markers of these pathways were confirmed by western blotting and RT‐qPCR analysis. These data provide a new level of understanding of the mechanistic role of miRNA to facilitate skeletal muscle adaptations to exercise with carbohydrate and protein feeding.

## Conflict of Interest

The authors have no conflicts of interests. The investigators adhered to the policies for protection of human subjects as prescribed in Army Regulation 70‐25, and the research was conducted in adherence with the provisions of 32 CFR part 219. The opinions or assertions contained herein are the private views of the authors and are not to be construed as official or as reflecting the views of the Army or the Department of Defense. Any citations of commercial organizations and trade names in this report do not constitute an official Department of the Army endorsement of approval of the products or services of these organizations. This study was approved by the Institutional Review Board at the US Army Research Institute of Environmental Medicine.
